# The WHO Safe Childbirth Checklist implementation: impact on the prescription of magnesium sulphate through a one-year longitudinal study

**DOI:** 10.1186/s12884-020-2836-z

**Published:** 2020-03-12

**Authors:** Zenewton André da Silva Gama, Wilton Rodrigues Medeiros, Pedro Jesus Saturno-Hernández, Kelienny de Meneses Sousa, Matheus Silva Mello, Érico de Lima Vale, Tatyana Maria Silva de Souza Rosendo, Edna Marta Mendes da Silva, Marise Reis de Freitas

**Affiliations:** 1 Department of Collective Health, Federal University of Rio Grande do Norte, Natal, Brazil; 2 Ana Bezerra University Hospital, Federal University of Rio Grande do Norte, Santa Cruz, Brazil; 3 National Institute of Public Health of Mexico (INSP), Avenida Universidad No. 655, Colonia Santa María Ahuacatitlán, C.P, 62100 Cuernavaca, Mor Mexico; 4 Graduate Program in Collective Health, Federal University of Rio Grande do Norte, Natal, Brazil; 5 Course of Medicine, Federal University of Rio Grande do Norte, Natal, Brazil; 6 Maternity School Januario Cicco, Federal University of Rio Grande do Norte, Natal, Brazil; 7 Infectious Diseases Department, Federal University of Rio Grande do Norte, Natal, Brazil

**Keywords:** Quality of health care, Patient safety, Hypertension, pregnancy-induced, Eclampsia, Preeclampsia, Magnesium sulphate

## Abstract

**Background:**

Preeclampsia is a relatively frequent condition during pregnancy and childbirth. The administration of magnesium sulphate as a prophylactic and treatment measure is an evidence-based practice for eclampsia; however, it is not consistently used, compromising the health of pregnant women. This study aimed to assess compliance with recommendations of the International Society for the Study of Hypertension in Pregnancy (ISSHP) for the use of MgSO_4_ in pregnant women with preeclampsia, before and after the implementation of the World Health Organization Safe Childbirth Checklist (SCC).

**Methods:**

This quasi-experimental study was conducted between July 2015 and July 2016 at a third-level maternity hospital in northeastern Brazil, where the SCC was implemented. Compliance (underuse and overuse of MgSO_4_) was assessed in biweekly samples of 30 deliveries assessed 6 months before and 6 months after SCC implementation, using indicators based on international guidelines. A total of 720 deliveries were assessed over 1 year using an ad hoc application for reviewing medical records. Aggregated adequate use was estimated for the study period, and the time series measurements were compared to a control chart to assess change.

**Results:**

The incidence of preeclampsia was 39.9% (287/720). Among these, 64.8% (186/287) had severe signs or symptoms and needed MgSO_4_. Underuse (no prescription when needed) of MgSO_4_ was observed in 74.7% (139/186) of women who needed the drug. Considering all women, non-compliance with the prescription protocol (underuse and overuse) was 20.0% (144/720). After introducing the SCC, the use of MgSO_4_ in women with preeclampsia with severe features increased from 19.1 to 34.2% (*p* = 0.025). Longitudinal analysis showed a significant (*p* < 0.05) ascending curve of adequate use of MgSO_4_ after the SCC was implemented.

**Conclusions:**

Compliance with recommendations for the use of MgSO_4_ in preeclampsia was low, but improved after implementation of the SCC. Interventions to improve compliance based on diagnosis and treatment reminders may help in the implementation of this good practice.

## Background

Hypertensive disorders of pregnancy (HDP) are the most frequent gestational complications, affecting around 10% of pregnant women worldwide [1]. They account for approximately 15% of maternal deaths in the United States, and about 25% in Latin America and the Caribbean [2]. In Brazil, the incidence of HDP in specific hospitals ranges from 14.4 to 18.4% [3, 4].

Preeclampsia (PE) is defined as a situation in which a previously normotensive pregnant woman becomes hypertensive, presenting proteinuria after the 20th week of pregnancy. However, the emergence of alarming signs, such as thrombocytopenia, kidney failure, visual or brain disorders (e.g., headache, scotomas or seizure), irrespective of the presence or absence of proteinuria, also indicates PE [5]. Eclampsia is characterised by the appearance of tonic-clonic seizures or coma in a pregnant woman with PE [5, 6]. A systematic review of studies including 39 million women across 40 countries estimated that PE and eclampsia occurred in 4.6 and 1.4% of all deliveries, respectively, although there was significant variability between regions [7].

Magnesium sulphate (MgSO_4_) is the recommended drug to prevent and treat eclampsia [1, 5, 6]. A number of studies have shown that MgSO_4_ reduced the risk of eclampsia by 58% in women with PE when compared to a placebo [8], and it was found to be more effective than other drugs that are currently in use [9, 10]. Thus, the drug is considered a good choice for prophylaxis against eclampsia in women with PE associated with severe complications (e.g., pulmonary edema, severe hypertension and acute kidney injury) [5]. In addition, it is a low-cost treatment that is well tolerated by patients [11, 12].

In 2011, the World Health Organization (WHO) published a guideline entitled “*WHO recommendations for prevention and treatment of pre-eclampsia and eclampsia”*, in which MgSO_4_ is identified as the drug of choice for treating and preventing eclampsia [1]. The International Society for the Study of Hypertension in Pregnancy (ISSHP) has made subsequent recommendations to this goal [5, 6]. The Brazilian guidelines use these same international recommendations [13]. However, it is not clear whether health professionals are following the recommendations and using MgSO_4_ properly.

The WHO Safe Childbirth Checklist (SCC) [14], currently adapted for Brazil [15], includes the use of MgSO_4_. At the site of this study, the SCC was implemented in 2014 [16]. However, the effect of SCC implementation on compliance with the MgSO_4_ protocol was not assessed.

The objective of this study is twofold: (1) assess adherence to the current MgSO_4_ guidelines, which is relevant by itself due to the relative lack of studies on this topic; and (2) evaluate to what extent the implementation of reminders such as the SCC may contribute to compliance with adequate use of MgSO_4_.

## Methods

### Context and design

The present study is part of a larger project that implemented the WHO SCC at two Brazilian maternity hospitals and five Mexican hospitals from 2015 to 2016 [15]. In the Brazilian facilities, this study monitored deliveries in 2-week periods from 6 months before to 6 months after implementation of the checklist. A public facility located in a state capital of northeastern Brazil was selected for this study. This facility is the state reference unit for high-risk deliveries, including care for women with PE. It has 141 beds, including six in the adult intensive care unit and 23 in the neonatal intensive therapy unit, performing an average of 12 deliveries per day and approximately 4300 per year. The other Brazilian maternity and Mexican institutions did not collect additional data on the use of MgSO_4_ in pregnant women with PE.

This study used a time series quasi-experimental design without a control group [17]. The SCC was implemented in the intervention facility, and data collected over the 6-month period after the intervention were compared to data from the 6-month period before the intervention. The fact that data collection occurred every 2 weeks for 1 year classifies it as a time series.

### Intervention

The intervention consisted of the implementation of the SCC in early 2015 [15]. During the period before the checklist was implemented, awareness was raised about the use of the checklist, followed by the assignment of responsibilities for completing the checklist and monitoring the implementation and feedback on adherence. The training of professionals was carried out through workshops, as well as the dissemination of an awareness video and instructional posters and brochures about its importance and correct use. This phase was developed by the professionals of the service with the support of the research promoting educational institution.

The checklist is composed of the following four phases: on admission, just before pushing (or before caesarean), soon after birth (within 1 h), and before discharge. Each of these phases includes a reminder of the indications and whether or not MgSO_4_ should be prescribed.

### Participants

The study population included women who gave birth during the study period. We considered the presence of newborn congenital anomalies as an exclusion criterion, to avoid biased results in the variables of interest. The sample consisted of 30 medical charts every 2 weeks for 1 year (total of 720). A sample size of 30 cases per measurement is considered sufficient to monitor the quality of health services using control charts [18]. The cases were selected by systematic random sampling from a list containing the date of hospitalisation within each 2-week period under study [19].

### Variables of interest

The variables of interest were the occurrence or not of PE, hospitalisation time, delivery type, mother’s age, the criterion that defined PE with severe features, compliance with the MgSO_4_ protocol, delivery before or after implementation of the SCC.

The variable related to the occurrence or not of PE was determined according to the 2014 ISSHP guideline [5], which defines PE as gestational hypertension, and one or more of the following: new proteinuria; one/more adverse condition(s); one/more severe complication(s). Adverse conditions are those associated to the risk of severe complications (headache/visual symptoms, chest pain/dyspnoea, oxygen saturation < 97%, elevated White Blood Cells count, elevated INR, low platelet count, elevated serum creatinine, elevated serum uric acid, nausea or vomiting, epigastric pain, elevated bilirubin, low plasma albumin, oligohydramnios, intrauterine growth restriction); severe complications are those that warrant delivery (eclampsia, cortical blindness, Glasgow coma scale < 13, stroke, oxygen saturation < 90%, positive inotropic support, myocardial ischaemia or infarction, platelet count < 50 × 10^9^/L, transfusion of any blood product, acute kidney injury, new indication for dialysis, hepatic dysfunction, hepatic haematoma or rupture, abruption, stillbirth). Severe preeclampsia is defined as preeclampsia with one or more severe complications [5]. We classified women with PE after measuring the variables gestational hypertension, proteinuria and all the adverse conditions and severe complications described above. Reverse ductus venosus A wave is a severe complication, but was not evaluated because this facility does not routinely perform this diagnostic practice [5].

Since ISSHP recommends prescribing MgSO4 for all women with severe features (systolic blood pressure ≥ 160 mmHg or diastolic blood pressure ≥ 110 mmHg, headaches / visual symptoms, right upper quadrant/epigastric pain, platelet count < 100,000 × 10^9^/L, progressive renal insufficiency, and/or elevated liver enzymes), we measured these clinical variables and classified women in need of MgSO4 following these criteria.

The use of MgSO_4_ was measured based on registered prescription on the medical chart assessed. Three general and complementary measures were estimated for the entire sample: (1) an overall indicator of compliance with the MgSO_4_ protocol measured by the number of cases when MgSO_4_ was prescribed in cases requiring it and when it was not prescribed in cases where it was not indicated; (2) overuse, measured as the number of cases where the drug was prescribed when the patient presented none of the criteria to justify the prescription; and (3) underuse, measured as the number of cases where the drug was not prescribed in cases that met the necessary criteria for prescription. In addition, adequate use (correct prescription) and underuse (not prescribed when needed) were separately evaluated in cases of PE.

### Data collection

Data were collected by nine undergraduate students in the health area, who had been previously trained and supervised by a doctoral student in the Public Health Graduate Program. An app was developed for data collection using tablets and online databank storage.

Before data collection, a pilot study with 30 medical charts was conducted to assess the reliability of the instrument, achieving adequate kappa indices (> 0.76). The pilot study cases were not included in this analysis. The complete dataset we have used for the analysis is provided as Additional file 1.

### Data analysis

The database was analysed using SPSS software (version 22) for cross referenced frequency reports and tables comparing maternal age, length of stay and caesarean rates for cases of PE and those without the disorder. Given the non-normal distribution of days of hospitalisation and maternal age variables, the Mann-Whitney test for two independent samples was applied to assess the statistical significance of differences between the two groups. The statistical significance of the differences regarding the type of delivery and the occurrence or not of PE was assessed using the chi-square test. All tests considered a 5% significance level, rejecting the null hypothesis of the differences when the *p*-value was ≤0.05.

With respect to analysis of the effect of SCC on compliance with the MgSO_4_ protocol, a control chart was constructed to observe trends and to assess significant changes in the compliance indicator, looking at the presence of pre-established patterns such as the presence of six or more consecutive ascending or descending points [18].

### Details of ethics approval

This study was approved by the Research Ethics Committee of Onofre Lopes University Hospital/UFRN on May 27, 2016 under protocol number 1,562,300/2016.

## Results

### Sample characteristics and criteria for preeclampsia

A total of 720 delivery charts were analysed, with 30 charts assessed every 15 days. In relation to the frequency of PE, 287 women met the criteria for PE (39.9%) according to the ISSHP [5]. Among these, 186 (64.8%) has PE with severe features and needed MgSO_4_. The exclusion criterion (presence of newborn congenital anomalies) was not present in any delivery, and no one was excluded for the analysis.

The length of stay and caesarean rate were significantly higher in women with PE (Table 1). The rate of caesarean delivery was also extremely high (60.5%) in women without PE. Of the women with PE, 71.4% delivered by caesarean. There was no intergroup difference in median maternal age. Forceps were used in only one delivery, involving a patient who did not meet any of the criteria for PE.

**Table 1:** Mean length of stay, average age and type of delivery of mothers with and without preeclampsia (PE) in a hospital in Rio Grande do Norte state, Brazil, in 2016

	PE (287/720)		Without PE (433/720)		***p***-value
**Variable**	Mean	Standard deviation	Mean	Standard deviation	
Length of stay (days)	4.5	4.7	3.2	1.7	0.000* 0.124
Age (years)	25.4	7.3	26.0	7.0	
**Variable**	Absolute value	Percentage	Absolute value	Percentage	
Vaginal delivery	82	28.6%	170	39.3%	0.007*
Caesarean delivery	205	71.4%	162	60.5%	
Forceps delivery	0	0.0%	1	0.2%	

The number of cases is presented in parentheses. *Variable with p ≤ 0.05

Among the criteria for PE with severe features [5], severe hypertension occurred in 76.9% of cases (143/186), low platelet count in 8.6% (16/186), and eclampsia in 3.2% (6/186) (Table 2).

**Table 2:** Main criteria used to establish preeclampsia (PE) with severe features (*n* = 186) in a hospital in Rio Grande do Norte, Brazil, in 2016

Variable*	Absolute value	Percentage
Severe hypertension (blood pressure of ≥160 mmHg systolic or > 110 mmHg diastolic)	143	76.9%
Platelet count < 100,000 × 10^9^/L	16	8.6%
Eclampsia	6	3.2%
Pulmonary oedema	4	2.2%
Oxygen saturation < 90%	3	1.6%
Stillbirth	3	1.6%
Transfusion of any blood product	3	1.6%
Abruption with evidence of maternal or foetal compromise	3	1.6%
Liver haematoma/rupture	2	1.1%
Intubation	2	1.1%
Need for positive inotropic agents	1	0.6%
Glasgow Coma Scale score < 13	1	0.5%
Hepatic dysfunction (International Normalized Ratio > 2)	0	0%
Creatinine > 1.7 mg/dl	0	0%
Need for haemodialysis	0	0%
Myocardial ischemia	0	0%
Stroke	0	0%
Cortical blindness	0	0%

*These criteria were determined according to the 2014 ISSHP guideline [5]

### Total compliance with the magnesium sulphate protocol over 1 year of follow up

Overall compliance with the MgSO_4_ protocol in the deliveries analysed was 80.0% (Table 3). The causes of noncompliance were not prescribed when needed (underuse, *n* = 139 patients) and prescribed when not needed (overuse, *n* = 5 patients). However, among the 186 patients who met the criteria for PE with severe features and need for MgSO_4_, the percentage of underuse was 74.7%. Therefore, MgSO_4_ was prescribed in about a quarter of women who needed the drug.

**Table 3:** Compliance with the prescription protocol for magnesium sulphate in all women and in those with preeclampsia (PE) with severe features before and after implementation of the Safe Childbirth Checklist in a hospital in Rio Grande do Norte, Brazil, in 2016

		TOTAL n = 720	BEFORE ***n*** = 360	AFTER n = 360	p-value
**Group of women**	**Compliance**	**% (number)**	**% (number)**	**% (number)**	
Overall (n = 720)*	**Yes**	80.0 (576)	74.7 (269)	85.3 (307)	0.001
	**No**	20.0 (144)	25.3 (91)	14.7 (53)	
**PE with severe features (n = 186)**	**Yes**	25.3 (47)	19.1 (21)	34.2 (26)	0.025
	**No**	74.7 (139)	80.9 (89)	65.8 (50)	

*“Yes” was recorded for an adequate prescription decision (prescription when needed or no prescription when not needed); “No” was recorded for an inadequate prescription decision (no prescription when needed or prescription when not needed)

### Changes in compliance in the magnesium sulphate protocol

**Fig. 1:**
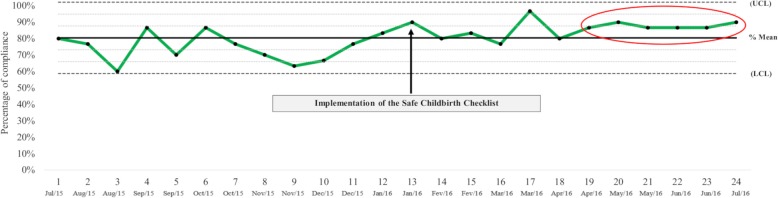
Compliance with the magnesium sulphate protocol every 2 weeks of the study period for all deliveries (*n* = 720) in 2016. *Six points above the average, indicating a significant improvement in the indicator. UCL, upper control limit; LCL, lower control limit. The black line represents the overall mean compliance with the MgSO_4_ protocol for all deliveries (83.2%). The green line represents the evolution of the percentage of compliance with the MgSO_4_ protocol

Compliance with the MgSO_4_ protocol in all deliveries increased from 74.7 to 85.3% (*p* = 0.001) after the implementation of SCC (Fig. 1). The control chart shows a significant increasing (*p* < 0.001) trend with six consecutive points (one point for every 2-week period) above the mean after implementation of the SCC.

Among patients with PE with severe features, compliance with the MgSO_4_ protocol increased from 19.1 to 34.2% after SCC implementation (*p* = 0.025).

## Discussion

This research estimated the frequency of hypertensive disorders of pregnancy in the assessed population and compliance with the MgSO_4_ protocol in all deliveries and in cases of PE with severe features, as well as the effect of implementation of the WHO SCC on improving adequate prescription of MgSO_4_. Our findings may have an impact on policies aimed to improve the quality and safety of maternal healthcare.

### Excessive rates of preeclampsia and caesarean delivery highlight the need for a more effective maternal health policy

The high frequency of PE observed in our study (39.9%) highlights the need to improve early diagnosis, preventive measures and treatment, given the increased risk of complications. Our study did not aim to evaluate the incidence of preeclampsia, but it is important to emphasise that the frequency of PE obtained in our study exceeds those reported in other studies and may need further careful evaluation. Systematic reviews have estimated that the prevalence of PE in low-income countries ranges from 1.8 to 16.7% [20] and from 2.7 to 8.2% globally [7]. A prospective study conducted in a community health unit from 2004 to 2006 in Natal, RN, reported a frequency of PE of 13.8% [21]. The higher rate found in the current study may be partially due to the method of data extraction: we directly reviewed medical records instead of diagnoses in the information system, which could be underestimated. In addition, it is important to consider the type of maternity hospital in which the study was conducted, which was a third-level facility that treats most cases of pregnancy-induced hypertensive disorders in the state, with an emphasis on women of lower socioeconomic status. Moreover, in low- and middle-income countries, the incidence of PE is higher than in high-income countries [22]. There is a significant difference in PE between low-income and high-income countries, representing the third direct cause of maternal death in the latter and the second in the former [2]. In our study, these differences seem to be even higher, underscoring the need to pay even greater attention to this maternal health problem.

High blood pressure was the most prevalent criterion to characterise PE with severe features (systolic blood pressure ≥ 160 mmHg and/or diastolic blood pressure ≥ 110 mmHg), which was present in 76.9% of the cases. This is similar to the findings of studies conducted in other countries [20], demonstrating the importance of accurate blood pressure measurement and monitoring in pregnant women [5].

We also found an extremely high rate of caesarean deliveries, which was even higher in women with PE (71.4% vs. 60.5%; *p* < 0.05). Similar caesarean rates in women with PE have been reported by other studies conducted in developing countries [23]. However, PE is not considered an absolute indication for caesarean delivery [13], and caesarean rates fluctuate greatly between and within countries [24]. The high rate found in the current study is probably not medically justified, and is not exclusively related to cases of PE. Indeed, Brazil presents high rates of caesarean section in general [24].

In other developing countries, the incidence of caesarean delivery in cases of PE is 42.6% [20], which is far below the frequency observed here. Furthermore, in a survey of PE practice patterns among members of the Society of Perinatal Obstetricians in the United States, the estimated overall caesarean delivery rate was 73% at 32 weeks of gestation in women with PE [25]. It is believed that caesareans are often performed electively rather than only when needed, which is also the case in PE. This situation must be improved to avoid the complications and costs inherent to this procedure. A recent population-based study in Brazil [26] found that, after adjusting for confounders and indications, the risk of postpartum maternal death was almost three-fold higher for caesarean than vaginal delivery, representing a serious health problem in a country that had an average caesarean delivery rate of 57% in 2014 [26]. The problem is likely to be worse in the context of our study, where caesarean delivery rates are well above the national average.

### Magnesium sulphate is underused despite evidence and national policies

MgSO_4_ was prescribed far less than expected (25.3%), a noticeable underuse. This finding is even more worrying if we consider that the study population is a group of very severe patients. This has also been observed in studies that explored the factors most commonly associated with the non-use of MgSO_4_ in other areas. These include a lack of specific institutional guidelines, equipment and trained personnel, a limited supply of the drug, in addition to the erroneous perception that its use is restricted to highly specialised contexts, such as intensive care units [11, 27].

Policies to reduce maternal mortality in Brazil include adequate management of hypertensive disorders of pregnancy. In this respect, one of the objectives of decree no. 399 of 2006 is to “guarantee supplies and medication to treat hypertensive syndromes of pregnancy” [28]. MgSO_4_ is on the National List of Essential Medication, which contains the standard drugs indicated to treat diseases in the National Health System and specifies its obstetric use in hospitals [29]. Thus, in Brazil, the law supports the use of MgSO_4_, as well as requiring that it be made available, even if only in hospitals. The lack of specific guidelines and personnel training are probably largely responsible for the low use of MgSO_4_ in the current study.

Other studies have found that an important barrier to the use of MgSO_4_ is the belief that it should be restricted to highly specialised centres and only used when the nursing team is trained to deal with complications [30]. However, current evidence suggests that concern about the adverse effects of MgSO_4_ is not justified, given that they occur in a small number of patients and are generally reversed by delaying administration of the next dose of the drug [31]. Thus, measures should be adopted to broaden the implementation of this medication, overcoming these barriers that preclude better maternal outcomes.

The overuse observed in a small number of cases may have occurred due to excessive use, but could also be attributed to some other factor not considered in this analysis, such as neuroprotection in cases of premature birth, which has been increasingly reported in the literature [32].

Overall compliance (adequate prescription decision) with the MgSO_4_ protocol in the whole sample was relatively high (80.0%). This includes prescribing MgSO_4_ when needed and not prescribing it when it is not indicated. However, the most serious problem is non-adherence to the protocol in those women who need MgSO4.

### The positive effect of implementing the WHO safe childbirth checklist

An important observation in our study was that compliance with the MgSO_4_ protocol improved significantly after implementation of the SCC. This result is in line with earlier studies which show that the SCC leads to the adoption of good practices during delivery [33, 34], and underscores the potential usefulness of checklists and reminders for quality improvement. Thus, the SCC should probably become a routine policy.

Although we identified a significant increase in MgSO_4_ use after SCC implementation, it was also modest. Probably, quality improvement projects with multifaceted interventions that include continuing education will help ensure greater impact [35].

### Limitations

A possible limitation of the present study is the use of medical records as the data source, given the shortcomings associated with these documents. In this regard, some PE cases may not have been detected, but it is unlikely that the prescription of MgSO_4_ and the caesarean section were not recorded. We did not explore the influence of parity and sociodemographic variables other than age. However, the main conclusions (high rates of PE and caesarean deliveries and underuse of MgSO_4_, particularly in severe PE cases) would remain unchanged.

## Conclusions

This study shows a high incidence of PE in the context assessed. This complication is associated with a longer length of hospital stay and higher rates of caesarean section. Although the use of MgSO_4_ is widely recommended for this condition when there are signs and symptoms of severity, it was found to be underused. On the other hand, during the 1-year follow-up, a significant improvement in the use of MgSO_4_ was observed after the SCC was implemented. This result suggests that focused reminders may favour the adoption of good practices, thereby preventing avoidable complications in pregnant mothers.

## Supplementary information

**Additional file 1.** Database preeclampsia and use of Safe Childbirth Checklist_Brazil. The database contains all cases and all variables used in this study.

## Data Availability

The datasets generated and/or analysed during the current study are available as Additional file 1.

## Abbreviations

MgSO_4_: Magnesium sulphate
PE: Preeclampsia
RN: Rio Grande do Norte (state of Brazil)
SCC: Safe Childbirth Checklist
WHO: World Health Organization
